# Characterization of Neural Network Connectivity and Modularity of Pigeon Nidopallium Caudolaterale During Target Detection

**DOI:** 10.3390/ani15040609

**Published:** 2025-02-19

**Authors:** Zhizhong Wang, Hu Wang, Juncai Zhu, Deyu Zhao, Rui Wang, Zhuangzhuang Ma, Shaoju Zeng, Jiangtao Wang

**Affiliations:** 1 School of Electrical and Information Engineering, Zhengzhou University, Zhengzhou 450001, China; 2 Beijing Key Laboratory of Gene Resource and Molecular Development, Beijing Normal University, Beijing 100875, China

**Keywords:** pigeon, target detection, nidopallium caudolaterale, neural network, decoding

## Abstract

Accurate target detection in natural environments is an important function of the visual systems of vertebrates and has a direct impact on animal survival and environmental adaptation. Existing studies have shown that the mammalian prefrontal cortex plays an important role in target detection. However, target detection mechanisms in brain regions similar to other species, such as the avian nidopallium caudolaterale, have not been well studied. Here, we selected pigeons, known for their excellent target detection ability, as an animal model and studied the dynamic changes in the nidopallium caudolaterale neural network features while they performed a target detection task in a maze. The results showed that the average node degree increased significantly during the target detection process while modularity decreased significantly. This indicated that functional connectivity in pigeon brains was enhanced during the task execution, the frequency of brain interactions increased, and the neural network shifted from distributed processing to more efficient integrated processing. The decoding results based on the average node degree and modularity and the combination of both showed that the accuracy of target decoding corresponding to the combination of both was higher. Taken together, our results confirmed the important role of the above properties for encoding target information. We provided evidence to support the view that the NCL is critical for target detection tasks and that studying key features of its neural network provides a powerful tool for revealing the functional state of the brain.

## Simple Summary

Efficient target detection is essential for animals to survive in complex and changing natural environments. Birds are particularly skilled at this ability, and a specific part of their brain, the nidopallium caudolaterale, plays a key role in detecting targets. Scientists often study this brain region to understand how birds detect targets. However, it is still unclear how network features of the nidopallium caudolaterale region change dynamically during this process. In this study, we used pigeons and placed them in a maze to explore changes in their neural network features before and after detecting a target. The results showed that when pigeons detected the target, the connections within their neural network features became stronger, and the network became less divided into separate modules. This suggests that pigeons shift their brain activity from widespread processing to a more efficient and focused mode when detecting targets. Our findings support the idea that the nidopallium caudolaterale brain region is crucial for target detection in birds and highlight the importance of its network features in processing target information. This research enhances our understanding of bird cognition and may benefit the development of artificial target detection systems, aiding fields such as robotics and artificial intelligence.

## 1. Introduction

Target detection is an important function of the visual system in animals and is crucial to their survival. Animals rely on their visual abilities to find food, identify enemies, and choose mates [1,2]. Studying the responses of specific brain regions during target detection not only helps to reveal the neural mechanisms of visual information processing but also deepens our understanding of animal behavior and cognition.

### 1.1. Studies of the NCL Brain Region in Birds

Relevant studies of mammals have shown that the prefrontal cortex (PFC) is able to optimize decision-making and behavior by modulating the activity of networks that predict and reflect target location and integrate environmental information throughout the target detection process. In a spatial navigation task in rats, neurons in the PFC guided their gradual approach to the target by encoding target location and path selection. The activity of the PFC increased significantly when the target was successfully detected [3]. In contrast, when the rats failed to detect the target, the activity level of their prefrontal cortexes decreased, suggesting that the activity of the PFC was closely linked to the successful detection of the target and the decision-making process [4].

In birds, the nidopallium caudolaterale (NCL), which has significant functional similarities to the mammalian PFC, is considered a crucial region involved in higher cognitive functions. It shows significant neural activity, especially during target search and detection [5,6]. When birds (e.g., pigeons) search for a target in a complex maze task, neuronal activity in the NCL increases significantly, peaking as they approach the target. This enhanced neural activity suggests that the NCL plays a vital role in integrating environmental information and detecting targets [7]. This suggests that the NCL is not only involved in the target detection and approach process but also significantly modulates its activity to facilitate decision-making and behavioral adjustment after target detection.

### 1.2. Studies of Neural Networks

Visual tasks rely on the cooperation of multiple brain regions or neurons. Studying the functional connectivity within and between different brain regions and their topology can provide key insights into the complex and dynamic information-processing mechanisms of the brain [8].

The features of neural networks include clustering coefficients, path lengths, the average node degree, modularity, and others. The clustering coefficient is a measure of the degree of connectivity between a node and its neighbors in a network. It indicates whether a node and its neighbors are connected. The path length refers to the shortest path between any two nodes in a network. It reflects the efficiency of information transfer between nodes in a network. The average node degree is a basic measure of the strength of connections between nodes in a network. It represents how many edges (connections), on average, each node in the network has compared to other nodes. Modularity describes the degree to which the network is divided into functional modules. It is used as a measure of how the nodes are grouped together to form communities or modules in the network [9,10].

Furthermore, whereas the features of neural networks maintain the basic functions of the brain in its resting state, they also adjust dynamically with different task demands, environmental changes, and cognitive loads. Studies have shown that when individuals face cognitive tasks or challenges, the clustering coefficients and path lengths of networks change to optimize the efficiency and reliability of information processing. The modular organization is also adjusted to balance local processing and global integration to support more complex cognitive functions [11,12].

In studies of mammals, especially humans and primates, network features play a key role in cognitive task performance. Functional connectivity between the PFC and other brain regions is enhanced when the human brain transitions from a resting state to the performance of cognitive tasks, suggesting that the brain maintains its functional integration and information-processing efficiency by strengthening connectivity between critical nodes [13]. In addition, primates show significant dynamic adjustments in their network features in the face of social stress or challenging tasks. The brain maintains cognitive and behavioral stability by modulating cortico-cortical connections and enhancing the synergy of functional networks [14]. In learning and memory tasks, specific regions in the brain’s functional networks exhibit enhanced plasticity, particularly a significant increase in the strength of network connectivity associated with memory encoding and retrieval to efficiently process information [15]. In target detection tasks, the brain needs to enhance functional connectivity between regions related to vision, memory, and decision-making by dynamically adjusting the network topology to ensure the accuracy and efficiency of target detection [16]. In most existing studies on pigeons, researchers have focused on visual cognition and behavioral responses. The mechanism of dynamic adjustments of their neural network during target detection tasks has not yet been explored in depth.

Here, to investigate the NCL information processing mechanism in pigeons during target detection, we designed a set of refined maze experiments. We analyzed the changes in the features of nidopallium caudolaterale neural networks (NNN) before and after the pigeons detected the target in the maze. Therefore, we aimed to reveal how the neural network changed in different cognitive states. Our study provided insights for understanding the mechanism by which information is processed in the NCL during target detection. We believe it can also provide inspiration for research on brain-like target detection algorithms in the field of machine vision.

## 2. Materials and Methods

### 2.1. Subjects

Four adult pigeons weighing 400–500 g were selected as experimental subjects, numbered 092, 106, 107, and 460. They were placed in separate pigeon cages and kept in an environment with 12 h of light and 12 h of dark cycles. They were allowed free access to water and fed food to maintain their weight within 80–85% of their free-feeding weight. All experiments were conducted in accordance with the requirements for the care and use of experimental animals under the 2006 Animal Law (China) and were approved by the Life Sciences Ethics Review Committee of Zhengzhou University.

### 2.2. Behavioral Apparatus and Protocol

**Figure 1:**
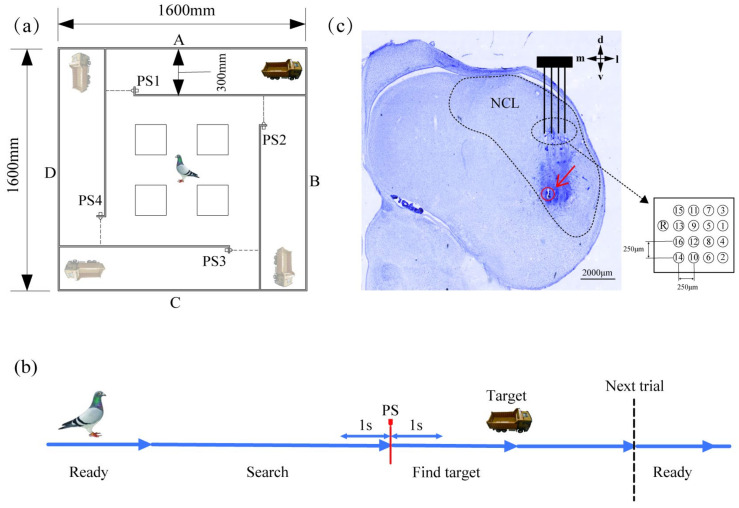
Behavioral paradigm, experimental flowchart, and electrode implantation sites. (**a**) The square maze consisted of four arms (A, B, C, and D). The photoelectric sensors (PS1, PS2, PS3, and PS4) were placed at the inlet of each arm to record the moment the pigeon enters the arm. Targets with rewards were placed at the end of the arm, and the target to be detected was randomly placed in one of the four arms in each trial, where the pigeons detected and approached the target to receive a food reward. (**b**) At the beginning of this experiment, pigeons searched for the target in the maze from the starting site. After searching the maze arm where the target was placed, they triggered the photoelectric sensor and entered the current arm, then approached the detected target and received a food reward. Then, they walked out of the current maze arm to start the next trial. (**c**) Histological identification of electrode implantation sites. The implantation site was electrically damaged using a timed DC negative pulse, and the microelectrode array was stained to obtain the implantation site. The red arrow indicates the specific electrode damage recording site.

As shown in Figure 1a, the experimental site was a rectangular labyrinth constructed using PVC boards (1.6 m long, 1.6 m wide, and 0.6 m high) with an exposed roof. The center of the labyrinth was set as the pigeon’s starting site at the beginning of the experiment, surrounded by a rectangular obstacle (20 cm long, 20 cm wide, and 60 cm high) surrounded by four arms (arms A, B, C, and D). Each arm was left with a 20 cm wide inlet. Photoelectric sensors (PS1, PS2, PS3, PS4) were installed at the inlets, and the pigeons triggered the photoelectric sensors every time they entered the arm where the target was placed. When the sensors were triggered, a timestamp of the triggering moment was recorded in the data acquisition and recording system, enabling the synchronization of behavioral events with electrophysiological signals during post-experiment data processing (the details are described in Supplementary Materials). A truck with a bucket was chosen as the target to be detected; the truck was randomly placed at the end of one of the four arms of the maze in each trial, and 6 g of food was placed in the bucket of the truck as a reward.

During the training phase of the target detection task, the pigeons were first placed at the start of the maze to search for the target in the maze. When it entered the maze arm where the target had been placed, the sensors at the inlet were triggered. The pigeon would then approach the target and be rewarded with food. After the pigeon fed and exited the maze arm, the target truck was replenished with food and placed randomly in a maze arm to begin the next trial (Figure 1b). Before the formal experiment, pigeons underwent pre-surgical and post-surgical training. During this period, pigeons were trained using the same paradigm as the formal experiment, and the total time required to complete the training was recorded each day. Pigeons were considered to have met the training requirements only if they completed at least 10 trials within 30 min on each of four consecutive training days. Pre-surgical training lasted for 9 days, with a one-day interval between each training day, spanning a total of 18 days. After surgery, pigeons rested for 7 to 10 days without any training tasks. Following recovery, pigeons underwent 5 days of post-surgical training, again with a one-day interval between each training day, spanning a total of 9 days. After the post-surgical training, pigeons rested for one day before beginning the formal experiment. Each pigeon participated in 6 days of the formal experiment, with a one-day interval between each experiment day, spanning a total of 11 days. No electrophysiological signals were collected during the pre-surgical and post-surgical training phases. Electrophysiological recordings began only at the start of the formal experiment phase. In the formal experiment, a trial was deemed invalid and excluded from subsequent analysis if the target was not correctly detected. During each trial, the pigeons spent approximately 2–3 min exploring the maze. The maximum exploration time for each trial was set to three times the average exploration time. If a trial exceeded this maximum time, it was also considered invalid. We have displayed the results of the behavioral data in the Supplementary Materials.

### 2.3. Surgery and Recordings

Before the surgery started, the pigeons were anesthetized with an injection of 3% sodium pentobarbital (0.2 mL/100 g body weight). Subsequent surgical procedures were performed under anesthesia. After anesthesia, the pigeons were immobilized on a custom-made stereotaxic apparatus, and 16-channel microneedle array electrodes (4 × 4 array, KD-MEA-16, KedouBC, Suzhou, China) were implanted in the left NCL brain region in conjunction with pigeon brain mapping [17]. A detailed illustration of the electrode channel distribution is shown in Figure 1c. The implantation sites of Bird 092 were ML: 7.5, AP: 5.5, and DV: 3.5. Those of Bird 106 were ML: 7.5, AP: 5.05, and DV: 3.75. Those of Bird 107 were ML: 7.5, AP: 5.05, and DV: 3.75. And those of Bird 460 were ML: 7.95, AP: 5.55, and DV: 2.4.

A Hermes Wireless Data Acquisition and Recording System (Jiangsu Yige Biotechnology Co., LTD, Nanjing, Jiangsu, China) was used to acquire neural signals. It is an advanced wireless data acquisition system that can amplify, filter, and digitize up to 64 channels of neural signals and store them in a microdata card with a sampling rate of 30 kHz. During this experiment, a camera (X5-1080p, S-YUE ShengYue, Yueqing City, China) fixed directly above the maze device was used to observe and record the pigeons’ experimental state, ensuring they were actively exploring the maze and engaged in the target detection task.

### 2.4. Histology and Electrode Track Reconstruction

When all data were collected, pigeons were anesthetized by injection of sodium pentobarbital (0.2 mL/100 g) at a concentration of 3%. Then, timed (30 s) DC (0.16 mA) negative-pulse electrical stimulation was used to injure the actual implantation sites of the electrodes. After this electrical injury, the animals were perfused using saline and fixative 4% paraformaldehyde. Brain tissues were then removed and fixed in a 4% paraformaldehyde solution for one day, then dehydrated in a 15% sucrose solution for one day, and finally dehydrated in a 30% sucrose solution for three days. The dehydrated pigeon brains were cryo-embedded and sectioned on a Leica cryosectioner at a thickness of 40 μm. Sections were dried in a thermostatic drying oven at 37 °C for 12 h. The sections were subjected to Nissl staining using standard histological protocols, and the staining results were observed and scanned using a VS200 research-grade whole-slide scanning system. The scanned images were analyzed with OlyVIA software (version V4.1), and the result was shown in Figure 1c.

### 2.5. Data Analysis and Processing

In this experiment, trials during which the pigeon detected the target and ate the food were considered valid. Data from 60 experimental trials per pigeon were used for subsequent analysis. The moments 1 s before and 1 s after the pigeon triggered the photoelectric sensor were used as the time window for the data analysis, with a 2 s period analyzed for each trial. The period 1 s before was identified as the pigeon not detecting the target, and the period 1 s after was considered as the pigeon finding and detecting the target.

**Figure 2:**
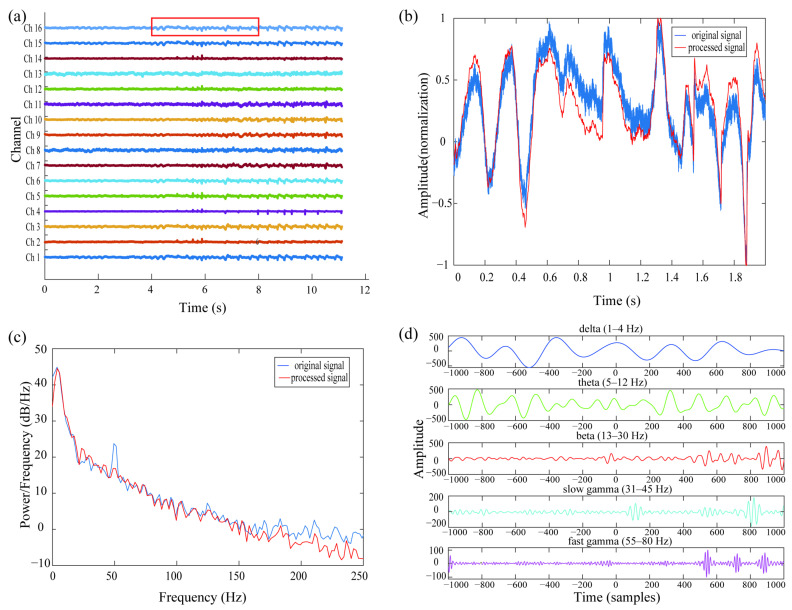
Preprocessing electrophysiological signals. (**a**) An excerpt of a 16-channel raw signal. (**b**) The original signal is shown in blue, and the signal obtained by low-pass filtering is shown in orange. The signal in the red box in (**a**) is used as an example of data processing. (**c**) Power spectrogram corresponding to the original signal (blue) and the signal after removing the power-frequency interference from it (red). Obvious power interference present at the 50 Hz band was eliminated. (**d**) The processed signal was band-pass filtered according to the delta (1–4 Hz), theta (5–12 Hz), beta (13–30 Hz), slow gamma (31–45 Hz), and fast gamma (55–80 Hz) bands to obtain waveforms in different frequency bands.

For the 16 channels of raw data collected by the acquisition device, 1–250 Hz LFP signals were filtered out from the raw signals with a Butterworth filter. Then, 50 Hz power-frequency interference was removed with the filter. The sampling rate was then downsampled from 30 kHz to 1 kHz. The processed signals were filtered according to the following five frequency bands: delta 1–4 Hz, theta 5–12 Hz, beta 13–30 Hz, slow gamma 31–45 Hz, and fast gamma 55–80 Hz (Figure 2) [18].

For each frequency band, data within the time window were intercepted, and the coherence of the signal was estimated by setting a sliding window with a window length of 256 samples (corresponding to 0.256 s) and a step size of 128 samples (corresponding to 0.128 s), using the multitaper method (MTM). The data in each window were calculated to obtain a 16 × 16 adjacency matrix containing the coherence data for each window. MTM is a nonparametric method for power spectrum estimation and is particularly suitable for dealing with non-stationary signals within short time windows. By using many window functions, MTM can effectively reduce the variance in spectral estimation, thus enhancing the stability and accuracy of spectral estimations [19]. Coherence, as an important measure of the degree of synchronization of two signals in the frequency domain, can reflect the phase consistency of two signals at a specific frequency [20]. For two signals, x(t) and y(t), their coherence $C_{xy}(f)$ at frequency f is defined as

(1) $$C_{xy}(f)=\frac{{\left|\langle S_{xy}(f)\rangle \right|}^{2}}{\langle S_{xx}(f)\rangle \langle S_{yy}(f)\rangle }$$

where $S_{xx}(f)$ and $S_{yy}(f)$ denote the self-power spectral densities of signals x(t) and y(t), respectively; $S_{xy}(f)$ is the cross power spectral density of signals x(t) and y(t), and 〈⋅〉 denotes the average for multiple superposition.

### 2.6. Threshold Selection

In the process of sparsifying the adjacency matrix, we are faced with the choice of threshold value. In order to avoid the limitations of using arbitrary thresholds in sparse adjacency matrices, we employ a robust, data-driven, percolation-based thresholding method. This method has been extensively validated in research [21,22,23].

To address the problem of selecting optimal thresholds when constructing sparse adjacency matrices from coherence data, we combine the percolation thresholding method and the Surprise method to select the optimal threshold for constructing the network. The Surprise method, as defined by Nicolini and Bifone [24], calculates the quality of network partitions using probability theory and has been shown to overcome the resolution limit, enabling the detection of small communities that are typically undetectable by traditional methods [25,26]. We define the threshold range as 0 to 1, evenly divided into 100 tiers. For each threshold, an adjacency matrix is constructed by retaining edges greater than that threshold, and the matrix is symmetrically processed to retain the upper and lower triangular elements. This iterative process progressively removes weaker connections and retains only the most robust functional relationships. At each threshold, we computed the Surprise metric, also known as the S-value, to assess the reliability of the detected community structure. By selecting the threshold corresponding to the maximum average value of the Surprise metric across all trials and birds within a frequency band, we determined the optimal threshold. This threshold effectively captures the most meaningful characteristics of network modularity and functional hierarchy while minimizing the influence of random fluctuations and resolution limitations [27,28,29,30]. In the subsequent section, we analyze the network features under the optimal threshold.

### 2.7. Average Node Degree and Module Degree

By constructing the coherence adjacency matrix, we can build neural networks. In neural network analysis, the average node degree and modularity are two important features commonly used to assess and understand the functional connectivity of the brain. The average node degree is the average value of the node degree of all the nodes in the network. It reflects the overall connectivity density of the network. The node degree describes the number of connections a node has to other nodes in the network, and the average node degree measures the average connection strength of the entire network [31]. Assuming that there are N nodes in the network and that the node degree of node i is $k_{i}$, the average node degree $\bar{k}$ is defined as

(2) $$\bar{k}=\frac{1}{N}\sum_{i=1}^{N}k_{i}$$

where $k_{i}$ denotes the degree of node i—that is, the number of other nodes to which the node is connected, and N is the total number of nodes in the network.

Modularity is a measure of the quality of the grouping of nodes (modules or communities) in a network. Modularity assesses the structural characteristics of the grouping of nodes in a network. High modularity indicates the presence of a pronounced modular structure in the network, where the nodes tend to be clustered into a number of tightly connected subsets with relatively little connectivity between the subsets [32].

Modularity Q is defined as

(3) $$Q=\frac{1}{2m}\sum_{i=1}^{N}\sum_{j=1}^{N}\left[A_{ij}-\frac{k_{i}k_{j}}{2m}\right]\delta (c_{i},c_{j})$$

where $A_{ij}$ is the adjacency matrix of the network, representing the connection between node i and node j; $\;k_{i}$ and $k_{j}$ are the node degrees of node i and node j, respectively; m is the total number of edges in the network, and $\delta (c_{i},c_{j})\;$ is an indicator function that is equal to 1 when i and j belong to the same module, and 0 otherwise. The value of modularity Q usually ranges between −1 and 1.

### 2.8. Decoding

In neuroscience research, decoding helps identify the response properties that represent specific stimuli or cognitive states. By analyzing the features of NNN, it is possible to assess the dynamic changes when the brain is processing target-related information. Through further decoding analysis, it is possible to identify the features of the network that most effectively reflect the target information. In this study, with ten-fold cross-validation, three decoders—support vector machine (SVM), long short-term memory (LSTM), and decision tree (DT)—were used for decoding the target information [33]. SVM is a supervised learning algorithm widely used in classification problems. The main goal was to find the optimal hyperplane to separate the samples of different classes and to maximize the interclass interval. It is particularly suitable for high-dimensional data classification tasks, such as decoding EEG signals or functional MRI data [34]. LSTM is a special kind of recurrent neural network designed for processing and predicting time series data. It is able to effectively capture long-term dependencies through its gating mechanism and solve the problem of gradient vanishing faced by traditional recurrent neural networks when dealing with long sequences [35]. DT is a tree-based classification algorithm that recursively divides data into different subsets, with each node representing a judgment condition for a feature and the leaf nodes corresponding to the final classification result. DT is intuitive and easy to explain, and it can deal with nonlinear feature relationships [36].

### 2.9. Statistical Analysis

We used the analysis of variance (ANOVA) test to compare the differences between the two sets of data. The level of significance was set at 0.05, which means that the results were considered statistically significant when the *p*-value was less than 0.05. Significance results are marked with asterisks, where “*” denotes *p* < 0.05, indicating that a significant difference was detected between the two sets of data, and “**” denotes *p* < 0.01, indicating that the difference was extremely significant. The above statistical analysis algorithms were written based on MATLAB software (version R2022b, Natick, MA, USA).

## 3. Results

### 3.1. Data Preprocessing

The preprocessing process of electrophysiological signals involved several key steps to improve the quality of the signal and reduce noise interference. First, the collected raw experimental data were processed using a Butterworth filter to perform filtering and remove powerline interference (Figure 2a). The main goal of this process was to remove high-frequency noise and 50 Hz power-frequency interference, which were usually significant and unavoidable in experimental environments. After filtering, the low-frequency signal was retained (Figure 2b). The original signal exhibited significant peaks of power-frequency interference in the 50 Hz band, whereas the power spectrogram of the processed signal showed that interference at the 50 Hz band was successfully eliminated, preserving the effective frequency components of the signal that were relevant to neural activity (Figure 2c).

Next, the processed signals were filtered according to different frequency ranges and divided into five main bands, as shown in Figure 2d: delta (1–4 Hz), theta (5–12 Hz), beta (13–30 Hz), slow gamma (31–45 Hz), and fast gamma (55–80 Hz). The figure shows the filtering results for each frequency band, and the signal characteristics in different frequency ranges can be clearly seen. In the low-frequency delta and theta bands, the signal fluctuated more slowly and with larger amplitude. As the frequency increased in the beta band, the signal fluctuated faster and with lower amplitude. In the slow gamma and fast gamma bands, the signal fluctuated more rapidly and with a smaller amplitude, which showed the characteristics of high-frequency neural activity.

### 3.2. Neural Network Construction

**Figure 3:**
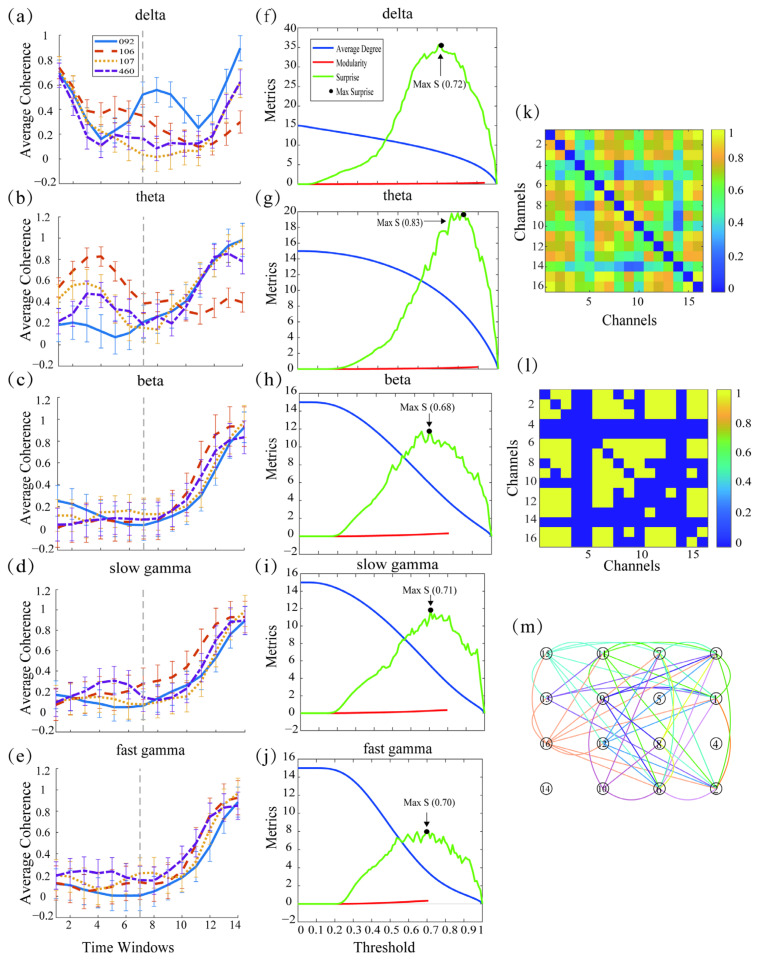
Channel coherence, threshold selection, and neural network composition. (**a**–**e**) Demonstration of temporal trends in global mean channel coherence for different bird species in different frequency bands. The dashed gray line in the figure represents the moment when the sensor is triggered. (**f**–**j**) The changes in the average node degree, modularity, and S-value of the neural network across different frequency bands with varying thresholds. Within each frequency band, the optimal threshold was determined by selecting the threshold corresponding to the maximum average value of S-value. (**k**) Schematic representation of the construction of an adjacency matrix based on inter-channel coherence. The color bar from blue to yellow indicates the magnitude of the coherence value, with yellow representing higher coherence and blue representing lower coherence. (**l**) Sparse matrix obtained after thresholding and binarizing the adjacency matrix using the optimal threshold. (**m**) The sparse matrix is visualized according to the electrode channel position, and the connecting lines illustrate the functional connectivity between different channels.

We next divided the data into five sub-bands. For each band signal, 1 s before and 1 s after the pigeon detects the target in each trial was intercepted and analyzed by a sliding window. The coherence between the channels was calculated and used to construct an adjacency matrix. Based on this, a neural network was constructed. Figure 3a–e shows the changes in the global channel coherence average across the sliding window for the four birds. In the beta, slow gamma, and fast gamma frequency bands, a significant increase in coherence values was observed after target detection compared to before target detection. In order to construct a clearer neural network that showed which connections in the brain may play a major role in information transfer or functional coordination, it was often necessary to sparsify the coherence matrix. As described in the Threshold Selection section, we adopted the Percolation thresholding method combined with the Surprise metric to determine the optimal threshold. Figure 3f–j illustrates the process of selecting the optimal threshold across different frequency bands. Additionally, Figure 3k–m demonstrates the process of neural network construction. Figure 3k shows an adjacency matrix based on inter-channel coherence, which is thresholded using the optimal threshold and then binarized to obtain the sparse matrix shown in Figure 3l. The sparse matrix is directly used for neural network construction, and its visualization is presented in Figure 3m, where the connecting lines between channels represent functional connections.

### 3.3. Average Node Degree and Modularity Analysis

**Figure 4:**
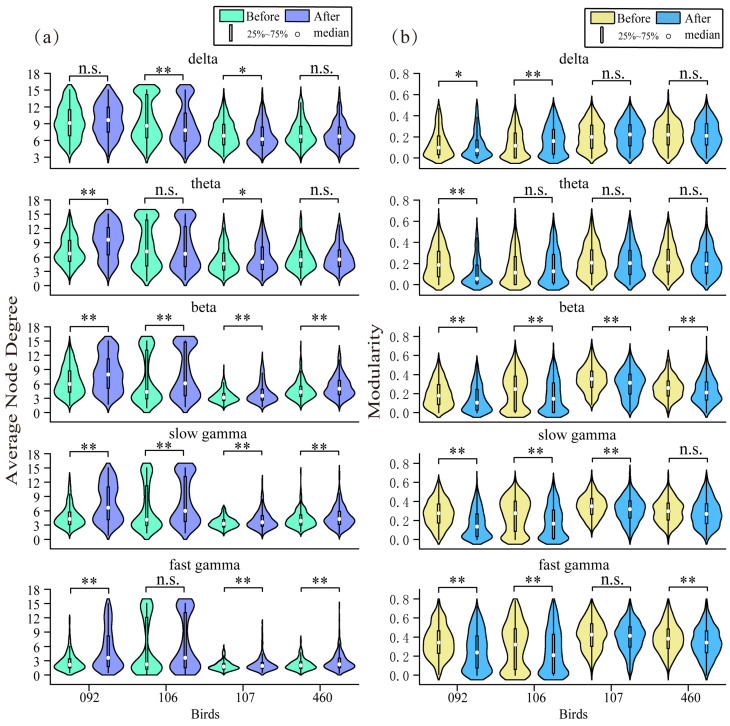
Violin plots of data analysis with and without detected targets in different frequency bands for two network features across four birds. (**a**) Data distribution of the average node degree before and after detecting the target in different frequency bands. In the beta and slow gamma bands, the average node degree before and after detecting the target significantly differed in each bird. (**b**) Data distribution of modularity before and after detecting the target in different frequency bands. In the beta band, the modularity before and after detecting the target was significantly different in each bird. ** *p* < 0.01, * *p* < 0.05, n.s. *p* < 0.01.

We analyzed the differences in the average node degree and modularity of the four birds in different frequency bands with and without detecting the target. We analyzed all the collected data, with each bird performing 60 trials, resulting in a total of 240 trials. The results of the neural network average node degree and modularity constructed based on the coherence of different frequency bands before and after each bird detected the target are shown in Figure 4a–b. Meanwhile, we present in Table 1 the mean ± standard deviation of the data for different birds in different frequency bands.

At the delta band, the average node degree of Bird 092 and Bird 460 showed no significant change before and after target detection (*p* = 0.1102 and *p* = 0.3316, respectively). The average node degree of Bird 106 significantly decreased (*p* = 0.0017), while that of Bird 107 slightly decreased (*p* = 0.0412). For modularity, the modularity of Bird 092 slightly decreased before and after target detection (*p* = 0.0417). Bird 106’s modularity significantly increased (*p* = 0.0023), whereas the average node degrees of Bird 107 and Bird 460 showed no significant changes (*p* = 0.1290 and *p* = 0.7223, respectively). These results indicate that in the delta band, the performance of different individuals showed variation.

At the theta band, the average node degree of Bird 092 increased significantly (*p* = 1.1068 × 10^−17^), indicating an increase in functional connectivity. The average node degree of Bird 107 slightly increased (*p* = 0.0216), while the average node degrees of Birds 106 and 460 showed no significant change (*p* = 0.3457 and *p* = 0.1763, respectively). Bird 092’s modularity decreased significantly, suggesting a decrease in network modularity despite an increase in average node degree (*p* = 1.0722 × 10^−19^). The modularity of Birds 106, 107, and 460 showed no significant change, indicating that the structure of their networks in the theta band was less affected (*p* = 0.3926, *p* = 0.5097 and *p* = 0.1914, respectively).

At the beta band, the average node degree significantly increased in all four birds (*p* = 1.6710 × 10^−12^, *p* = 0.0017, *p* = 3.1845 × 10^−8^ and *p* = 4.0111 × 10^−6^, respectively), suggesting that this band was very sensitive to targets and generally enhanced functional connectivity. In contrast, modularity at the beta band significantly decreased in all four birds (*p* = 1.2868 × 10^−8^, *p* = 1.3843 × 10^−6^, *p* = 1.1189 × 10^−8^, and *p* = 7.9529 × 10^−4^, respectively). In general, this implied that the brain at this moment required greater integration and flexibility, with increased collaboration between multiple, otherwise independent functional modules and a greater tendency for functional networks to integrate in order to process and respond to goal-relevant information more efficiently. This change supported the rapid transfer and integration of information across modules [37].

At the slow gamma band, the average node degree increased significantly in all four birds (*p* = 4.4265 × 10^−35^, *p* = 6.1456 × 10^−5^, *p* = 3.7965 × 10^−8^ and *p* = 4.5607 × 10^−4^, respectively), indicating that this band was also very sensitive to targets, with enhanced functional connectivity and increased information transfer when targets are present. Modularity decreased significantly in Birds 092, 106, and 107 (*p* = 1.9267 × 10^−27^, *p* = 5.1030 × 10^−8^, and *p* = 4.2595 × 10^−5^, respectively), showing that the modular structure of the network was weakened in these birds. The modularity of Bird 460 did not change much compared to them (*p* = 0.0646).

At the fast gamma band, the average node degree of Birds 092, 107, and 460 significantly increased (*p* = 3.1284 × 10^−27^, *p* = 4.6208 × 10^−5^ and *p* = 2.0118 × 10^−7^, respectively). The average node degree of Bird 106 showed little change compared to the others (*p* = 0.0544). For modularity, the modularity of Birds 092, 106, and 460 significantly decreased (*p* = 3.4478 × 10^−14^, *p* = 5.4107 × 10^−4^ and *p* = 1.3174 × 10^−4^, respectively). The modularity of Bird 107 showed little change compared to the others (*p* = 0.0882).

Overall, at the beta and slow gamma, the average node degrees of the neural networks of all four birds showed significant differences before and after detecting the targets. The distribution intervals of the average node degree increased significantly, indicating that after detecting the target, the functional connectivity of brain areas significantly increased. However, only at the beta band did the average node degree and neural network modularity of all four birds show significant differences. The distribution interval of modularity decreased significantly, indicating that the modular structure of the network weakened after detecting the target despite the increase in connectivity. This may reflect the shift of the functional neural network toward higher integration and cross-regional collaboration.

### 3.4. Decoding Analysis

According to the analysis in the previous section, the average node degree and modularity of the four pigeons under the beta band were significantly different before and after target discovery. To verify the importance of these features in representing and detecting target-related information and to evaluate their decoding performance in target detection, we constructed the dataset using the network features in the beta band from all four pigeons. We constructed three types of decoding matrices based on network features: node degree, modularity, and their combination. For each pigeon, we first extracted the node degree and modularity features separately from the data. Each feature matrix had dimensions of 120 × 7, where 120 represented the number of trials (60 trials per pigeon, divided into pre- and post-target detection), and 7 represented the number of time windows. Then, we concatenated the node degree and modularity features column-wise to generate a combined feature matrix, resulting in a matrix with dimensions of 120 × 14. Finally, we appended the label vector to each feature matrix, then combined the feature matrices of all four birds to obtain the final decoding matrix, and decoded it using three decoders: SVM, LSTM, and DT.

**Figure 5:**
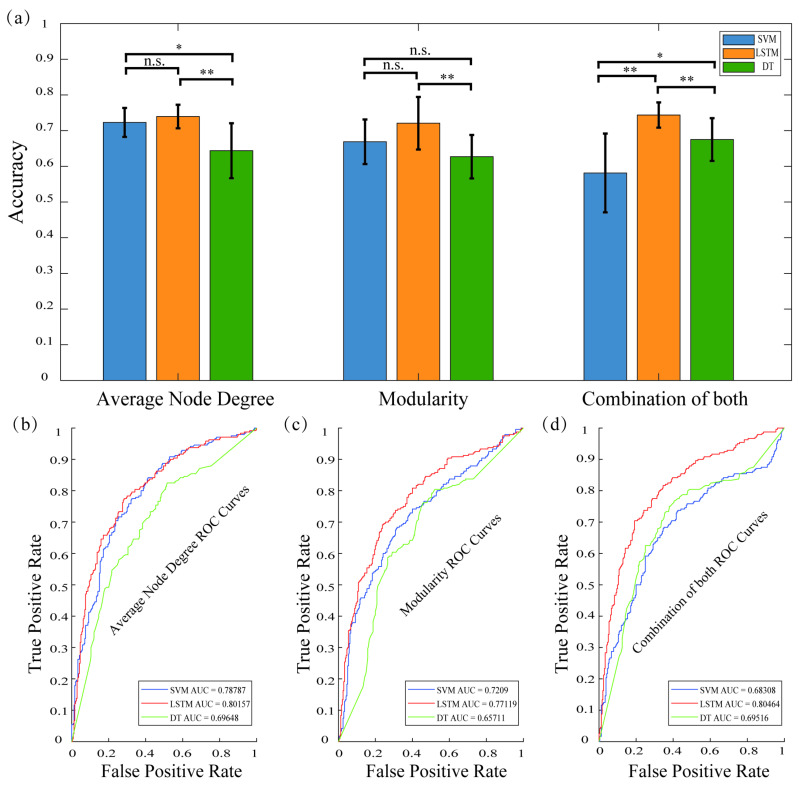
Decoding results of beta-band data. (**a**) The target was decoded based on two neural network features and the combination of both, with three decoders: SVM, LSTM, and DT. The LSTM decoder performs better than the other two decoders on all three features. (**b**) ROC curve for decoding based on the average node degree. (**c**) ROC curve for decoding based on the modularity. (**d**) ROC curve for the decoding based on the combination of average node degree and modularity. The AUC of LSTM was significantly better than that of other models, especially when decoding based on the combination of two neural network features, where it had the best classification ability. ** *p* < 0.01, * *p* < 0.05, n.s. *p* < 0.01.

The decoding results are shown in Figure 5a, which shows the decoding accuracies of the three decoders (SVM, LSTM, and DT) on the three features (average node degree, modularity, and the combination of both); the decoding accuracies of all results exceed 0.5, exceeding the baseline level. Additionally, compared to the accuracy of the other two decoders, all three features achieved the highest decoding accuracy with the LSTM decoder, demonstrating that the LSTM decoder performed significantly better than the other two decoders for our data. Furthermore, the decoding accuracy of the combination of both features with the LSTM decoder was higher than the accuracy of the other two features with the LSTM decoder, indicating that the LSTM model can more effectively capture the dynamic changes in neural networks by utilizing multiple features. Specifically, for the average node degree, the decoding accuracy of the LSTM was slightly higher than that of the SVM, with similar performance and no significant difference (*p* = 0.3264). However, significant differences were observed between SVM and DT (*p* = 0.0100), as well as between LSTM and DT (*p* = 0.0020). For modularity, there were no significant differences between SVM and LSTM or DT (*p* = 0.1052 and *p* = 0.1486, respectively), but there was a significant difference between LSTM and DT, with the LSTM decoder clearly outperforming the DT decoder (*p* = 0.0061). For the combination of both features, the results of all three decoders showed differences pairwise. Significant differences were observed between SVM and LSTM (*p* = 3.2322 × 10^−4)^, as well as between LSTM and DT (*p* = 0.0298), with both SVM and DT performing worse than LSTM. Additionally, SVM and DT also exhibited differences (*p* = 0.0059).

We drew receiver operating characteristic (ROC) curves of the three decoders on different feature sets and derived the corresponding area under the ROC curve (AUC) values (Figure 5b–d). The AUC was used to quantify the classification performance of the decoders. The closer the value was to 1, the better the classification performance. For average node degree, SVM had an AUC of 0.78787, which showed better classification performance. LSTM had an AUC of 0.80157, which was similar to SVM and showed similar classification performance. DT had an AUC of 0.69648, which was lower than SVM and LSTM, indicating that its classification performance was relatively poor. For the modularity, the AUC of LSTM was 0.77119, which was better than SVM (0.7209) and DT (0.65711), showing that LSTM had the best classification performance when modularity was used. For the combination of both, LSTM had the best performance, with an AUC of 0.80464. DT had an AUC of 0.69516, which was better than SVM but not as good as LSTM. SVM had an AUC of 0.68308, which was significantly lower than both LSTM and DT, showing that SVM had poorer classification performance on the combination of both.

The effectiveness of these neural network features in the target detection task was verified by decoding and analyzing the average node degree, the modularity, and the combination of both. Comparing the different decoding models, the accuracy and AUC of LSTM on the combination of both were significantly higher than those of the SVM and DT, suggesting that the NNN may represent target information in a temporally structured manner.

## 4. Discussion

In this study, we adopted an experimental paradigm for a target detection task in a maze to investigate the neural mechanisms of the NCL region in pigeons when performing a target detection task. By analyzing the dynamic changes in the features of NNN, we found that a neural network constructed based on the coherence of channels in the beta frequency band clearly reflected the differences in the state of pigeons before and after detecting a target. The experimental results showed that the average node degree of the network constructed based on this frequency band increased significantly, and the modularity decreased significantly after the pigeons detected the target. This finding suggested that the functional connectivity of the NCL mediated through the beta band was significantly enhanced during target detection in pigeons. Furthermore, the overall density of the neural network increased, reflecting more interaction and collaboration among the various regions of the NCL. This enhanced integration may allow the entire network to become more unified, contributing to more efficient processing of target-related information.

These results suggested that pigeons may undergo a significant functional reorganization of their NCL region when performing target detection tasks. From the perspective of the experimental paradigm, our study provided a novel target detection task by designing a maze behavioral experiment to reveal the dynamic changes in a neural network during target detection by pigeons. Compared with traditional visual stimuli or decision-making tasks, our maze experiment better simulated the complex decision-making process in the natural state of animals, and it provided a window of observation for understanding the information-processing mechanisms of the NCL during target detection [38].

From the perspective of neural network features, our study had two important findings. First, the average node degree significantly increased during the target detection task. This implied more interactions and increased functional connectivity among the different regions of the brain. Thus, during target detection, the brain needed to mobilize more resources to integrate information processing. This was similar to results found in other studies, such as van den Heuvel and Sporns, who showed that as the brain performed complex tasks, the increase in average node degree reflected more efficient information flow and integration in neural networks [39]. Second, we found that during target detection, modularity decreased, implying that the boundaries between functional modules became less distinct after the brain detected a target. This was perhaps due to enhanced collaboration between different brain regions to support more complex cognitive functions. This is consistent with the research of Bassett and Bullmore, who found that as cognitive load increased, the modular structure of the brain tended to disintegrate, and neural networks showed more global integration [40]. Decreased modularity, in turn, implied that the division of labor between individual brain regions was no longer strict and that the boundaries between functional modules became blurred. As such, the brain was shifting from distributed processing to more highly integrated processing to ensure rapid information transfer and processing.

From the perspective of decoding, based on the neural network features in the beta band, we used three decoders to classify and analyze the brain signals during target detection. By analyzing the decoding of the average node degree, modularity, and the combination of both, we further validated the importance of these features in representing and detecting target-related information. Although all decoders used the same neural network features, the decoding results differed. Compared to the other two decoders, the LSTM decoder, which excels at processing time-series features, performed better. That is, LSTM decoded target information more effectively, indicating that temporal dynamics in the neural network may play a crucial role in representing target information during detection tasks.

There are many fields worth further exploration based on this experimental paradigm. For instance, studies in the reptilian visual cortex have demonstrated that spindles and ripples play crucial roles in visual processing [41]. Investigating whether similar phenomena occur in the pigeon brain is an intriguing avenue for future research. Moving forward, we aim to analyze dynamic changes in band power within the NCL region during target detection, such as the temporal evolution of beta-band power, to uncover the dynamic characteristics of neural activity. Additionally, examining oscillatory features—such as band power fluctuations or the average coherence across all channel pairs—could not only enhance neural signal decoding frameworks but also deepen our understanding of the neural mechanisms underlying target detection. Furthermore, while target positions in this study were randomly distributed across trials, future investigations into the potential effects of target position on band power distribution could provide valuable insights into the role of spatial variables in target detection. These research directions promise to build on the current findings and offer a more comprehensive and nuanced theoretical foundation for understanding neural activity in target detection tasks.

There were limitations to our study. First, the relatively small sample size may have affected the breadth of the results. Future studies should expand the sample size to verify the general applicability of our findings. In addition, for each frequency band, we mainly focused on the beta band in our analysis. Future studies should explore the role of different frequency bands for target detection, especially in complex tasks where the interactions between different frequency bands may reveal deeper neural network dynamics. In addition, our experiment only analyzed the NCL. Although the NCL played an important role in target detection and high-level cognitive tasks, other regions in the brain, such as the visual cortex and the parietal cortex, may also have played key roles in the target detection process. Finally, although we used a variety of decoders to analyze brain signals, there were limitations regarding the algorithm selection and parameter settings.

Future studies should focus on exploring the interactions of different frequency bands of signals in target detection tasks. In particular, different frequency bands may reflect different levels of information processing. In addition, increasing the sample size in the experiment and introducing signal analysis for other relevant brain regions will help clarify the functional changes in the pigeon brain during complex cognitive tasks. Future studies could also explore the dynamic changes in the NCL during other cognitive tasks (e.g., learning, memory, and decision-making) to reveal the diversity and complexity of cognitive functions in birds. Finally, applying the methodology of this study to other species will help elucidate the similarities and differences in cognitive functions among different species and provide new perspectives for understanding the development of cognitive functions in brain evolution.

## 5. Conclusions

In this study, the features of NNN during a target detection task were analyzed. The results showed that the functional connectivity of the NCL significantly enhanced during target detection, and the functional network shifted from distributed processing to more efficient integrated processing. In addition, target decoding experiments highlighted the significance of the average node degree and modularity in representing target information. The LSTM decoder effectively captured complex temporal dynamics in the data. Our study provided a richer understanding of the cognitive functions of birds and contained new ideas for broader cognitive neuroscience research. In future studies, the dynamics of NNN when performing different tasks will be explored, laying a foundation for understanding the mechanisms of complex cognitive functions.

## Figures and Tables

**Table 1:** Mean ± standard deviation of average node degree and modularity.

Indicators	Bands	State	Bird 092	Bird 106	Bird 107	Bird 460
Average node degree mean ± std	delta	before	9.35 ± 2.96	9.59 ± 3.77	7.18 ± 2.33	7.24 ± 2.31
		after	9.67 ± 2.89	8.79 ± 3.59	6.85 ± 2.36	7.09 ± 2.22
	theta	before	7.34 ± 2.88	8.32 ± 4.71	5.36 ± 2.73	5.78 ± 2.55
		after	9.28 ± 3.51	8.02 ± 4.52	5.82 ± 3.04	6.03 ± 2.63
	beta	before	6.60 ± 2.97	6.94 ± 5.07	3.40 ± 1.35	4.73 ± 1.99
		after	8.28 ± 3.77	8.03 ± 4.97	4.09 ± 2.13	5.41 ± 2.26
	slow gamma	before	4.78 ± 2.27	6.59 ± 4.78	3.46 ± 1.10	4.24 ± 1.76
		after	9.63 ± 3.91	7.93 ± 4.80	4.05 ± 1.88	4.73 ± 2.21
	fast gamma	before	2.73 ± 1.92	5.67 ± 5.67	1.95 ± 1.00	2.22 ± 1.22
		after	5.28 ± 4.25	6.42 ± 5.66	2.33 ± 1.61	2.79 ± 1.87
Modularity mean ± std	delta	before	0.14 ± 0.13	0.14 ± 0.13	0.21 ± 0.13	0.22 ± 0.12
		after	0.12 ± 0.12	0.17 ± 0.14	0.22 ± 0.12	0.22 ± 0.13
	theta	before	0.21 ± 0.15	0.16 ± 0.16	0.23 ± 0.15	0.23 ± 0.14
		after	0.11 ± 0.13	0.17 ± 0.16	0.22 ± 0.15	0.22 ± 0.14
	beta	before	0.20 ± 0.13	0.24 ± 0.19	0.36 ± 0.13	0.26 ± 0.11
		after	0.14 ± 0.14	0.18 ± 0.17	0.30 ± 0.15	0.23 ± 0.14
	slow gamma	before	0.28 ± 0.14	0.25 ± 0.17	0.35 ± 0.11	0.30 ± 0.12
		after	0.17 ± 0.15	0.19 ± 0.17	0.31 ± 0.13	0.28 ± 0.14
	fast gamma	before	0.35 ± 0.17	0.31 ± 0.24	0.42 ± 0.15	0.39 ± 0.15
		after	0.26 ± 0.20	0.25 ± 0.22	0.40 ± 0.15	0.35 ± 0.16

## Data Availability

The data that support the findings of this study are available on request from the corresponding author.

## Supplementary Materials

The following supporting information can be downloaded at: https://www.mdpi.com/article/10.3390/ani15040609/s1, Figure S1: The behavioral data from the pre-surgical training, post-surgical training, and formal experiments.
