# Supplementary material for: Characterization of Neural Network Connectivity and Modularity of Pigeon Nidopallium Caudolaterale During Target Detection
Source: Animals (Basel). 2025 Feb 19;15(4):609. doi: 10.3390/ani15040609 (PMC11852068; doi:10.3390/ani15040609)
Supplement: Supplementary file 1 [file animals-15-00609-s001.zip › animals-3333976-supplementary.pdf]

Supplementary Materials

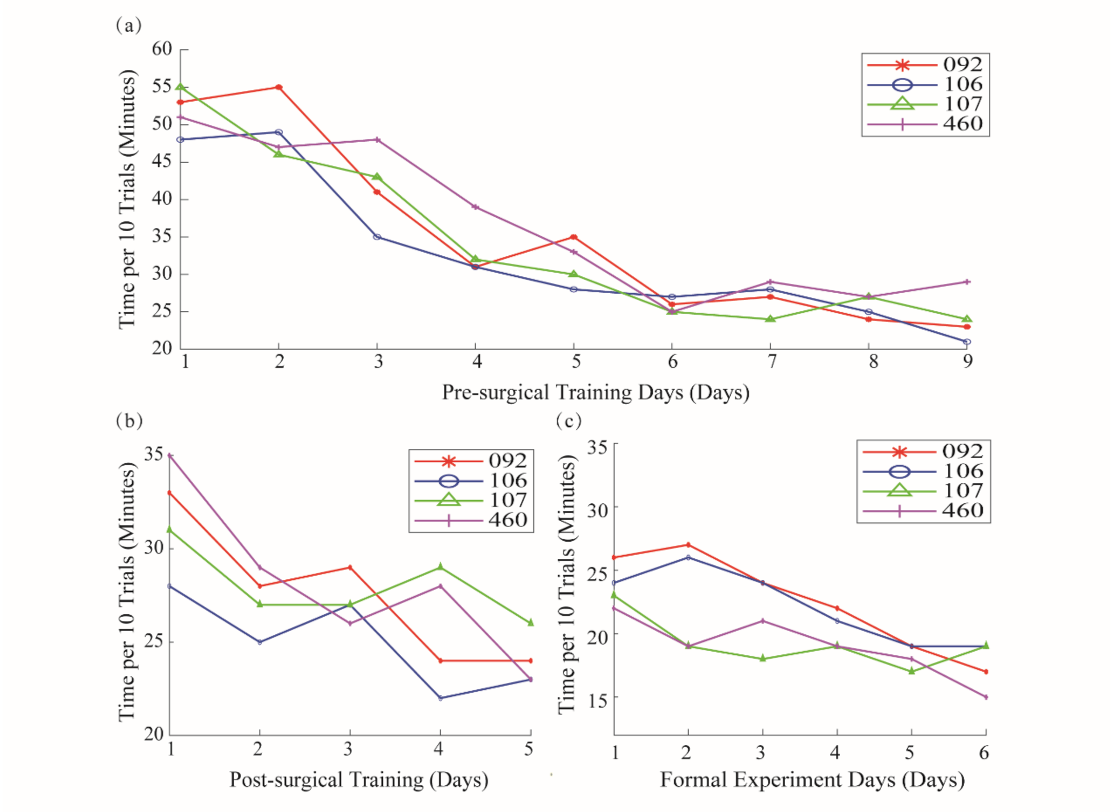

Figure S1. The behavioral data from the pre-surgical training, post-surgical training, and formal experiments.

The synchronization of behavioral events with electrophysiological signals:

In our experiment, the Hermes wireless data acquisition system's miniature headstage was attached to the pigeon's head to collect electrophysiological signals. These signals were stored in BSPF format on the headstage's internal memory card. However, the headstage itself could not record the exact moments when the pigeon triggered the sensor (PS) in the maze.

To address this, the sensors in the maze were connected to a Siemens S7-200 SMART PLC. Using the STEP 7-Micro/WIN SMART software, we programmed the PLC so that when the pigeon triggered a sensor, the PLC would output a 5V signal to the Hermes data acquisition system's marker board. This event created a timestamped entry in the log file of the host computer. After the experiment, we combined the BSPF file (containing electrophysiological data) and the log file (containing sensor trigger timestamps) in the Hermes software to produce a PLX file. This PLX file included both the neural signals and the corresponding timestamps of sensor triggers. We then analyzed this PLX file in MATLAB, thus achieving synchronization between behavioral events and electrophysiological data.
